# Significance of genetic variants in *DLC1* and their association with hepatocellular carcinoma

**DOI:** 10.3892/mmr.2015.3970

**Published:** 2015-06-22

**Authors:** CHENG-RONG XIE, HONG-GUANG SUN, YU SUN, WEN-XIU ZHAO, SHENG ZHANG, XIAO-MIN WANG, ZHEN-YU YIN

**Affiliations:** Department of Hepatobiliary Surgery, Zhongshan Hospital, Xiamen University, Fujian Provincial Key Laboratory of Chronic Liver Disease and Hepatocellular Carcinoma, Xiamen, Fujian 361004, P.R. China

**Keywords:** deleted in liver cancer 1, single nucleotide polymorphism, hepatocellular carcinoma, mutation

## Abstract

*DLC1* has been shown to be downregulated or absent in hepatocellular carcinoma (HCC) and is associated with tumorigenesis and development. However, only a small number of studies have focused on genetic variations of *DLC1*. The present study performed exon sequencing for the *DLC1* gene in HCC tissue samples from 105 patients to identify functional genetic variation of *DLC1* and its association with HCC susceptibility, clinicopathological features and prognosis. A novel missense mutation and four non-synonymous single nucleotide polymorphisms (SNPs; rs3816748, rs11203495, rs3816747 and rs532841) were identified. A significant correlation of rs3816747 polymorphisms with HCC susceptibility was identified. Compared to individuals with the GG genotype of rs3816747, those with the GA (odds ratio (OR)=0.486; P=0.037) or GA+AA genotype (OR=0.51; P=0.039) were associated with a significantly decreased HCC risk. Furthermore, patients with the GC+CC genotype of rs3816748, the TC+CC genotype of rs11203495 or the GA+AA genotype of rs3816747 had small-sized tumors compared with those carrying the wild-type genotype. No significant association of *DLC1* SNPs with the patients' prognosis was found. These results indicated that genetic variations in the *DLC1* gene may confer a risk for HCC.

## Introduction

Hepatocellular carcinoma (HCC) is one of the most common cancers worldwide, with >600,000 mortalities per year, 55% of which are in China (1). Risk factors of HCC include hepatitis B (HBV) or-C infection, high alcohol intake, smoking and nonalcoholic fatty liver disease (2). HCC is increasingly becoming a serious health problem in China. Recent studies have focused on finding somatic mutations in HCC by whole-exome sequencing. The results of these studies have shown that HCC is associated with somatic mutations in several genes, including *TP53*, *ARID1A*, *IRF2*, *MLL4* and *CTNNB1* (3–5). However, these studies did not fully elucidate the underlying mechanisms of the carcinogenesis and occurrence of HCC.

Deleted in liver cancer 1 (*DLC1*), which is located on chromosome 8p22, was first mapped in 1998 by Yuan *et al* (6). *DLC1* consists of 18 exons. As a tumor suppressor gene, the protein encoded by *DLC1* can regulate the structure of the actin cytoskeleton and inhibit cell proliferation, migration, invasion and angiogenesis (7–9). *DLC1* has been shown to be downregulated in several cancer types, including HCC, breast cancer, nasopharyngeal cancer and cervical cancer (10). Promoter hypermethylation, low-level acetylated H3 and H4 and enriched H2K27me3 are responsible for silencing *DLC1* expression (11–13). Besides the epigenetic silencing mechanisms of *DLC1* in HCC, little is known about the association of *DLC1* variants with HCC. Single nucleotide polymorphisms (SNPs) are the most frequent genetic variations in the human genome. Epidemiological studies have demonstrated that genetic variants are involved in various phases of carcinogenesis, which may determine susceptibility to the development of HCC (14,15). The synonymous SNP rs621554 of *DLC1* was reported to be significantly associated with HBV–associated HCC (16). However, the non-synonymous SNPs of *DLC1* that typically alter the gene product by changing the amino acid sequence of the protein have not yet been investigated in association with HCC. The present study aimed to determine the association of non-synonymous SNPs of *DLC1* with HCC susceptibility, clini-copathological features and prognosis in a Chinese population.

## Materials and methods

### Ethics statement

The present study was approved by the ethics committee of Xiamen Zhongshan Hospital (Xiamen, China). All subjects provided written informed consent.

### Study subjects

The present study analyzed 105 patients with HCC at Xiamen Zhongshan Hospital (Xiamen, China). Diagnosis was confirmed based on histological examination of the specimens. The specimens were transferred to a −80°C freezer immediately after surgery for long-term storage until analysis. Patient pathology information was obtained from pathology reports.

### Measurement of α-fetoprotein (AFP)

Serum samples were obtained from the above-mentioned patients. Serum AFP was measured by microchip capillary electrophoresis and a liquid-phase binding assay on a µTAS Wako i30 Auto Analyzer (Wako Pure Chemical Industries, Ltd., Osaka, Japan). All processes were performed automatically and followed the manufacturer's instructions.

### Sequencing exons of DLC1

To identify functional genetic variation of *DLC1*, the whole coding region was sequenced in all of the 105 HCC patients' samples. DNA was extracted from frozen tissue specimens using the TIANamp Genomic DNA kit (Tiangen Biotech, Beijing, China). The *DLC1* exome sequencing for HCC samples was performed by BGI (Guangzhou, China).

### Statistical analyses

All statistical analyses were performed using SPSS version 19.0 for Windows (International Business Machines, Armonk, NY, USA). χ^2^ tests or Fisher's exact tests were used to determine the associations among the *DLC1* genotypes, HCC risk and clinicopathological characteristics. The strength of association between polymorphisms and HCC risk was assessed by calculating odds ratios (ORs) with the corresponding 95% confidence intervals (CIs). The Kaplan-Meier method with a log-rank test was used to establish whether the *DLC1* SNPs influence the prognosis of HCC patients. All tests were two-sided, and P-values less than 0.05 were considered to indicate statistically significant differences.

## Results

### Exome sequencing of DLC1

**Figure 1:**
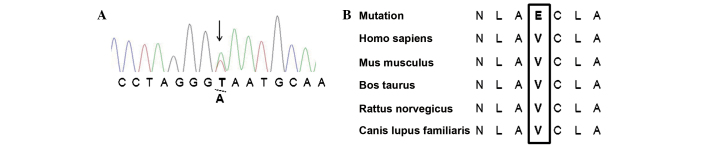
(A) Sequence analysis shows nucleotide sequence with a novel heterozygous missense T→A mutation at 3,743 in *DLC1*. (B) The 1,248th amino acid of the DLC1 protein in different species.

The present study identified genetic variants within the coding region of *DLC1* via exome sequencing. Previously reported mutations in *DLC1* were not identified in the subjects of the present study (17). However, in one patient, a novel heterozygous missense mutation (T→A) in exon 14 at nucleotide position 3,743 was identified (Fig. 1A). This mutation resulted in a change in the amino acid from Val to Ile at codon 1,248, which is in the Rho GAP domain. The 1,248th amino acid of the DLC1 protein is highly conserved among a variety of species (Fig. 1B).

For analysis, non-synonymous SNPs with the ability to affect encoded protein function were selected. A total of four non-synonymous SNPs were identified. The features of the non-synonymous SNPs of *DLC1* in HCC patients are shown in Table I.

### Association of DLC1 SNPs with HCC susceptibility

A total of 197 records of healthy Chinese Han individuals from the 1,000 Genomes database were used as a control group (http://www.ncbi.nlm.nih.gov/variation/tools/1000genomes/). The association between each of the *DLC1* genotypes and the risk of HCC is shown in Table II. A significant correlation of rs3816747 polymorphisms with HCC susceptibility was identified. Compared to individuals with the GG genotype of rs3816747, those carrying the GA (OR=0.486; 95%CI=0.245–0.962; P=0.037) or GA+AA (OR=0.51; 95%CI=0.267-0.974; P=0.039) genotype were associated with a significantly decreased risk of HCC. However, no significant differences were observed in the frequency distribution of rs3816748, rs11203495 and rs532841 between the case and control groups.

### Association of DLC1 SNPs with clinicopathological characteristics of HCC

To further determine the clini-copathological significance of *DLC1* SNPs, an univariate analysis was performed by using χ^2^ tests or Fisher's exact tests to correlate the genotypes of these four non-synonymous SNPs with clinicopathological features (Table III). Of note, a significant association of rs3816748, rs11203495, rs3816747 and tumor size was revealed. Patients with a GC+CC genotype of rs3816748, TC+CC genotype of rs11203495 or genotype GA+AA of rs3816747 had small-sized tumors compared with those of wild-type genotype patients. However, these *DLC1* SNPs had no significant association with gender, portal vein tumor thrombus, AFP and tumor differentiation level. Patients with a GC+CC genotype of rs3816748, TC+CC genotype of rs11203495 or GA+AA genotype of rs3816747 had small-sized tumors compared with those of wild-type genotype patients.

### Association of DLC1 SNPs with HCC prognosis

**Figure 2:**
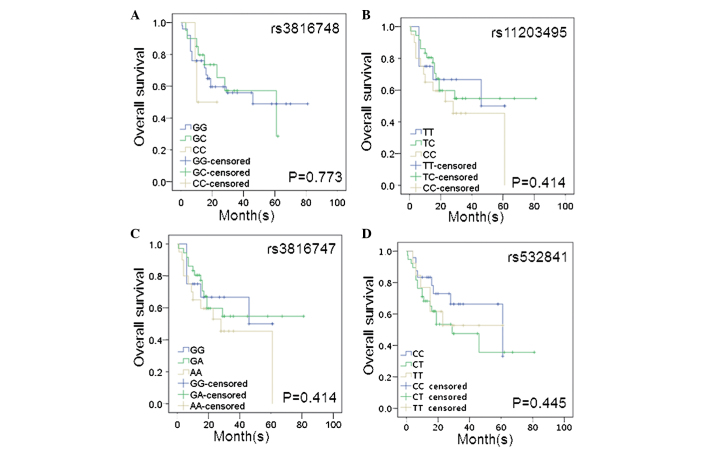
Survival analysis of *DLC1* single nucleotide polymorphisms in hepatocellular carcinoma based on genotypes. (A) Kaplan-Meier curves of overall survival for rs3816748 (GG, 50 individuals; GC, 20; and CC, 4), (B) rs11203495 (TT, 16; TG, 36; and TT, 20), (C) rs3816747 (GG, 16; GA, 36; and AA, 20) and (D) rs532841 (CC, 24; CT, 38; and TT, 13) genotypes in *DLC1*.

**Figure 3:**
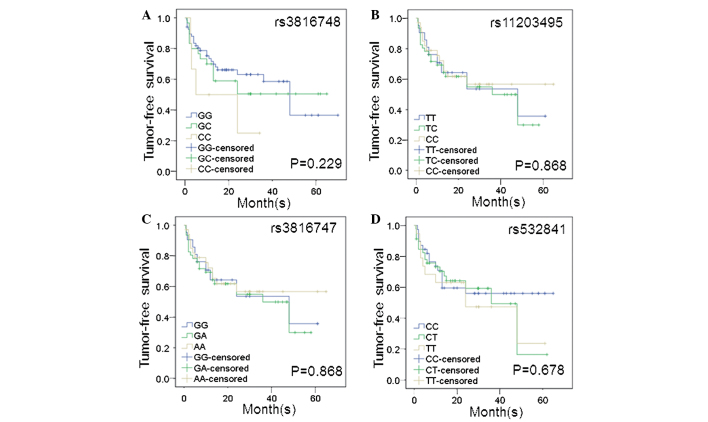
Survival analysis of *DLC1* single nucleotide polymorphisms in hepatocellular carcinoma based on genotypes. (A) Kaplan-Meier curves of tumor-free survival for rs3816748 (GG, 67 individuals; GC, 30; and CC, 6), (B) rs11203495 (TT, 21; TG, 46; and TT, 34), (C) rs3816747 (GG, 21; GA, 46; and AA, 34) and (D) rs532841 (CC, 39; CT, 46; and TT, 19) genotypes in *DLC1*.

The prognostic impact of *DLC1* SNPs was assessed by using Kaplan-Meier survival curves and the log-rank test (Fig. 2). The patients were followed every three months subsequent to surgery until the follow-up deadline, January 2013, was reached. A total of 42.1% of the patients had succumbed to HCC at the end of the follow-up period. However, none of the *DLC1* SNPs was associated with the overall survival rate. In addition, the association of tumor-free survival rates with the *DLC1* SNPs was assessed. Similarly, no significant differences in tumor-free survival between different genotypes of *DLC1* were observed (Fig. 3).

## Discussion

The present study identified a novel missense mutation and four non-synonymous SNPs in *DLC1* in HCC samples via exome sequencing. A study by Park *et al* (17) reported that in 17 primary HCC tumor samples and 18 HCC cell lines, only one missense mutation (Val991Ile) in *DLC1* was detected, namely in the START domain in the Hep40 cell line. However, this mutation was not detected in the subjects of the present study. *DLC1* mutation was rare and was only identified in one patient in the study. The novel mutation which was identified in the present study was located in exon 14, which encodes the Rho GAP domain. According to the pathological report of this patient, the tumor showed poor differentiation. This patient also had a shorter survival time (2 months) than the median survival time (61 months). Rho guanine triphosphatase (GTPase) activity is frequently deregulated in human cancers, and is involved in actin cytoskeleton remodeling, migration, metastasis, cell proliferation, transcription and tumorigen-esis (18,19). The multiple function of DLC1 mainly depends on the Rho GAP domain, which catalyzes the conversion of an active GTP-bound Rho to the inactive guanine diphos-phate-bound form (20). Therefore, DLC1 is able to inhibit tumorigenic and metastatic processes. Further functional analysis demonstrated that this mutant lost its tumor suppressive ability (data not shown).

A comprehensive genotyping analysis of a Chinese population provided evidence that a *DLC1* SNP was associated with susceptibility to HBV-associated HCC (16). This SNP was located in intron 19 of *DLC1*, which may influence the transcription of *DLC1* by changing the binding sites for a number of transcription factors or the target site of microRNA (16). However, the present study focused on non-synonymous *DLC1* SNPs in HCC patients. A significant association between rs3816747 polymorphisms and susceptibility to HCC was observed. The variant genotype GA+AA of rs3816747 conferred a 0.51-fold decreased susceptibility to HCC. It was also found that the variant genotypes GC+CC of rs3816748, TC+CC of rs11203495 and GA+AA of rs3816747 were significantly associated with decreased tumor size. It was therefore hypoth-esized that the non-synonymous SNPs that change the amino acid sequence of the protein may alter the function of the DLC1 protein. The *DLC1* gene encodes four isoforms, which occur due to a heterogeneity in promoters and alternative splicing at the 3′-end (20). The rs3816748, rs11203495, and rs3816747 polymorphisms are all located in exon 2 of *DLC1*. Exon 2 encodes the N-terminal region before the SAM domain, which only exists in isoform 1 and isoform 3 (21). However, the function of this region has remained to be fully elucidated. It has been reported that DLC1 isoform 1 is less efficient in exerting its tumor-suppressive activity compared with DLC1 isoform 2, which lacks the N-terminal region (22). In the present study, it was therefore hypothesized that this region may alter the tumor growth suppressive effect in an auto-inhibitory manner through interaction with other proteins, or by changing its localization. These non-synonymous SNPs in exon 2 change the amino acid sequence of the N-terminal region, which may decrease the auto-inhibitory effect. Further functional study of the N-terminal region and genetic variants will be necessary to reveal the molecular mechanisms underlying the association between non-synonymous SNPs of *DLC1* and HCC.

Previous studies have reported associations between SNPs in certain genes and HCC prognosis, including rs2640908 in *PER*, rs1741981 in *HDAC1* and rs2547547 in *HDAC3* (23,24). However, in the present study, no signifi-cant association of *DLC1* SNPs with prognosis was found. Similarly, Ko *et al* (22) reported that DLC1 expression was not associated with prognosis. Although restoration of DLC1 resulted in the inhibition of colony formation, cell migration and invasion *in vitro* as well as reduction of the development and metastasis of tumors *in vivo* and *in vitro* (25), DLC1 did not influence the prognosis in HCC patients. Recently, Roessler *et al* (26) identified survival-associated driver genes by conducting an integrative genomic profiling analysis of 76 patients with HBV-associated HCC. Six tumor suppressor genes, including *DLC1* on chromosome 8p, were found to be deleted in patients with poor prognosis (26). These studies suggested that *DLC1* SNPs on their own do not have any important role in HCC prognosis.

In conclusion, mutations of *DLC1* in the HCC patients examined in the present study were rare. The rs3816747 polymorphism in *DLC1* had a predictive value for HCC susceptibility in a Chinese population. Three SNPs (rs3816748, rs11203495 and rs3816747) in exon 2 were associated with tumor size. However, these SNPs were not associated with prognosis and are therefore not suitable as prognosis markers. To the best of our knowledge, the present study was the first to investigate the association of all non-synonymous SNPs of *DLC1* found in HCC patient samples with HCC susceptibility, clinicopathological characteristics and prognosis in Chinese subjects. However, a larger population and further exploration of the molecular mechanisms of *DLC1* SNPs may be required for further confirmation of this association.

## Figures and Tables

**Table I:** Characteristics of nonsynonymous SNPs of *DLC1*.

SNP ID	Allele	Exon	AA	AA position
rs3816748	G/C	2	Leu/Val	81
rs11203495	T/G	2	Gln/His	254
rs3816747	G/A	2	Thr/Ile	260
rs532841	C/T	9	Val/Met	791

SNP, single nucleotide polymorphism; AA, amino acid.

**Table II:** Genotypic analyses of *DLC1* single nucleotide polymorphisms and their association with HCC susceptibility in a Chinese population.

Genotype	HCC, n (%)	1,000 GENOMES, n (%)	OR	95% CI	P-value
rs3816748					
GG	68 (65.38)	111 (56.35)	1 (reference)	–	–
GC	30 (28.85)	77 (39.09)	0.636	0.379–1.068	0.086
CC	6 (5.77)	9 (4.57)	1.088	0.371–3.192	0.878
GC+CC	36 (34.62)	86 (43.65)	0.683	0.418–1.118	0.129
rs11203495					
TT	21 (20.59)	38 (19.29)	1 (reference)	–	–
TC	47 (46.08)	101 (51.27)	0.842	0.446–1.590	0.596
CC	34 (33.33)	58 (29.44)	1.061	0.537–2.095	0.865
TC+CC	81 (79.41)	159 (80.71)	0.922	0.508–1.674	0.789
rs3816747					
GG	21 (20.59)	23 (11.68)	1 (reference)	–	–
GA	47 (46.08)	106 (53.81)	0.486	0.245–0.962	**0.037**
AA	34 (33.33)	68 (34.52)	0.548	0.266–1.126	0.1
GA+AA	81 (79.41)	174 (88.32)	0.51	0.267–0.974	**0.039**
rs532841					
CC	39 (37.14)	75 (38.07)	1 (reference)	–	–
CT	47 (44.76)	89 (45.18)	1.016	0.601–1.715	0.954
TT	19 (18.10)	33 (16.75)	1.107	0.559–2.195	0.861
TC+TT	66 (62.86)	122 (61.93)	1.04	0.638–1.697	0.874

Bold values are statistically significant (P<0.05). OR, odds ratio; 95% CI, 95% confidence interval; HCC, hepatocellular carcinoma.

**Table III:** Correlation between *DLC1* single nucleotide polymorphism genotypes and clinicopathological characteristics in study subjects.

Genotypes	Gender			Tumor size			PVTT			AFP			Differentiation			
	Male n (%)	Female n (%)	P	<5 cm n (%)	≥5 cm n (%)	P	No n (%)	Yes n (%)	P	<400 n (%)	≥400 n (%)	P	Low n (%)	Medium n (%)	High n (%)	P
rs381674																
GG	57 (67.06)	11 (57.89)		12 (48.00)	51 (63.75)		23 (65.71)	37 (64.91)		40 (71.43)	19 (55.88)		6 (46.15)	50 (68.49)	5 (55.56)	
GC	23 (27.06)	7 (36.84)	0.824	11 (44.00)	15 (18.75)	0.083	10 (28.57)	16 (28.07)	1	13 (23.21)	14 (41.18)	0.099	7 (53.85)	18 (24.66)	3 (33.33)	0.226^a^
CC	5 (5.88)	1 (5.26)		2 (8.00)	14 (17.50)		2 (5.71)	4 (7.02)		3 (5.36)	0 (0.00)		0 (0.00)	5 (6.85)	1 (11.11)	
GC+CC	28 (32.94)	8 (42.11)	0.448	13 (52.00)	29 (36.25)	**0.024**	12 (34.29)	20 (35.09)	0.937	16 (28.58)	14 (41.18)	0.182	7 (53.85)	23 (31.51)	4 (44.44)	0.280
rs11203495																
TT	14 (16.87)	6 (33.33)		2 (8.00)	19 (27.54)		5 (14.29)	14 (24.56)		15 (26.79)	4 (12.50)		1 (7.692)	17 (23.94)	1 (11.11)	
TC	41 (49.40)	6 (33.33)	0.304^a^	13 (52.00)	28 (40.58)	0.133	18 (51.43)	24 (42.11)	0.466	27 (48.21)	15 (46.88)	0.169	4 (30.77)	31 (43.66)	6 (66.67)	0.159
CC	28 (33.73)	6 (33.33)		10 (40.00)	22 (31.88)		12 (34.29)	19 (33.33)		14 (25.00)	13 (40.63)		8 (61.54)	23 (32.39)	2 (22.22)	
TC+CC	69 (83.13)	12 (66.67)	0.196	23 (92.00)	50 (72.46)	**0.045**	30 (85.71)	43 (75.44)	0.237	41 (73.21)	28 (87.50)	0.117	12 (92.31)	54 (76.06)	8 (88.89)	0.368
rs3816747																
GG	14 (16.87)	6 (33.33)		2 (8.00)	19 (27.54)		5 (14.29)	14 (24.56)		15 (26.79)	4 (12.50)		1 (7.692)	17 (23.94)	1 (11.11)	
GA	41 (49.40)	6 (33.33)	0.304^a^	13 (52.00)	28 (40.58)	0.133	18 (51.43)	24 (42.11)	0.466	27 (48.21)	15 (46.88)	0.169	4 (30.77)	31 (43.66)	6 (66.67)	0.159
AA	28 (33.73)	6 (33.33)		10 (40.00)	22 (31.88)		12 (34.29)	19 (33.33)		14 (25.00)	13 (40.63)		8 (61.54)	23 (32.39)	2 (22.22)	
GA+AA	69 (83.13)	12 (66.67)	0.196	23 (92.00)	50 (72.46)	**0.045**	30 (85.71)	43 (75.44)	0.237	41 (73.21)	28 (87.50)	0.117	12 (92.31)	54 (76.06)	8 (88.89)	0.368
rs532841																
CC	35 (40.70)	4 (21.05)		11 (44.00)	22 (30.99)		16 (43.24)	20 (35.09)		26 (46.43)	11 (32.35)		5 (38.46)	27 (36.49)	3 (33.33)	
CT	39 (45.35)	8 (42.11)	0.056	9 (36.00)	35 (49.30)	0.448^a^	17 (45.95)	22 (38.60)	0.187	20 (35.71)	16 (47.06)	0.410	4 (30.77)	32 (43.24)	6 (66.67)	0.401
TT	12 (13.95)	7 (36.84)		5 (20.00)	14 (19.72)		4 (10.81)	15 (26.32)		10 (17.86)	7 (20.59)		4 (30.77)	15 (20.27)	0 (0.00)	
CT+TT	51 (59.30)	15 (78.95)	0.109	14 (56.00)	49 (69.01)	0.239	21 (56.76)	37 (64.91)	0.427	30 (53.57)	23 (67.65)	0.188	8 (61.54)	47 (63.51)	6 (66.67)	1

a Fisher's exact test. P<0.05 are given in bold. (P-values indicate the following: rs381674, compared with GG; rs11203495, compared with TT; rs3816747, compared with GG and rs532841, compared with CC). AFP, alpha-fetoprotein; PVTT, portal vein tumor thrombus; P, P-value.
